# Time since faecal deposition influences mobilisation of culturable *E*. *coli* and intestinal enterococci from deer, goose and dairy cow faeces

**DOI:** 10.1371/journal.pone.0274138

**Published:** 2022-09-02

**Authors:** Emmanuel O. Afolabi, Richard S. Quilliam, David M. Oliver

**Affiliations:** Faculty of Natural Sciences, Biological & Environmental Sciences, University of Stirling, Stirling, United Kingdom; University of California Davis, UNITED STATES

## Abstract

Mobilisation is a term used to describe the supply of a pollutant from its environmental source, e.g., soil or faeces, into a hydrological transfer pathway. The overarching aim of this study was to determine, using a laboratory-based approach, whether faecal indicator bacteria (FIB) are hydrologically mobilised in different quantities from a typical agricultural, wildlife and wildfowl source, namely dairy cattle, red deer and greylag goose faeces. The mobilisation of FIB from fresh and ageing faeces under two contrasting temperatures was determined, with significant differences in the concentrations of both *E*. *coli* and intestinal enterococci lost from all faecal sources. FIB mobilisation from these faecal matrices followed the order of dairy cow > goose > deer (greatest to least, expressed as a proportion of the total FIB present). Significant changes in mobilisation rates from faecal sources over time were also recorded and this was influenced by the temperature at which the faecal material had aged over the course of the 12-day study. Characterising how indicators of waterborne pathogens are mobilised in the environment is of fundamental importance to inform models and risk assessments and develop effective strategies for reducing microbial pollution in catchment drainage waters and associated downstream impacts. Our findings add quantitative evidence to support the understanding of FIB mobilisation potential from three important faecal sources in the environment.

## Introduction

Faecal pollution of surface waters, commonly measured by the presence of faecal indicator bacteria (FIB), can be linked to a variety of catchment sources. These include wastewater discharge points and combined sewer overflows, runoff from agricultural land and contributions from wildlife and wildfowl [1–4]. FIB delivered to receiving waters via point sources such as effluent pipes are mobilised and transferred through a combination of managed water flows and engineered infrastructure. In contrast, FIB contributed from agricultural and wildlife/wildfowl sources are mobilised and transferred largely as a function of rainfall-runoff responses in the environment [5]. The latter represent a supply of FIB that are distributed across the landscape as diffuse sources associated with direct faecal deposits or applications of manures and slurries to land.

The level of faecal pollution contributed from diffuse sources is related to a variety of factors, e.g., the burden of FIB in the environment (source factors; [6]), landscape and environmental characteristics that influence the generation of runoff (transfer factors; [7]) and the extent of hydrological connectivity linking hillslope to stream which provides a pathway for FIB being transferred in runoff (delivery factors; [8]). The crucial step that supplies a pollutant from its source into the hydrological transfer pathway is termed mobilisation [9]. The mobilisation of FIB from source material distributed across a landscape is largely driven by detachment processes. For example, the physical disruption of faeces by raindrop impact can dislodge faecal particles and FIB, whilst the resulting overland flow following rainfall also has the potential to slough FIB from faecal sources [10–12].

The occurrence of a rainfall event therefore provides an energy source to physically disrupt the faecal deposit and initiate mobilisation, and rainfall characteristics such as intensity and volume have been investigated as determinants of microbial water quality [13]. The importance of factors such as angle of sloping land and how this varies at different scales, e.g., plot to hillslope, in influencing rainfall-induced release of FIB and their subsequent loss from land to water have also been explored [11,14]. However, those studies have focused on quantifying FIB after their transfer across a soil surface and subsequent delivery to a receptacle or receiving water but have not specifically quantified all FIB mobilised from the faecal matrix. There can be significant increases in post-rainfall FIB numbers in soil, which highlights that although FIB can be released from faeces during rainfall, not all of these FIB are necessarily transferred to a receiving water in a single rainfall event, e.g., [15]. In one study where mobilisation was quantified directly, a laboratory assay was used to determine relative differences in FIB release rates from sheep and beef cow faeces, in addition to dairy slurry and beef cow manure [16]. The methodology of [16] modified a protocol originally used to measure phosphorus mobilisation from soil under highly controlled conditions and mimicked the impact of a rainfall event, providing important data on differences in FIB mobilisation attributed to faecal substrate and FIB type. Data concerning the mobilisation potential of FIB from a range of faecal types, and not just livestock sources, can be used to better parameterise process-based models of FIB fate and transfer in environmental systems, e.g., [17], or to support and inform more simple risk-based approaches to mapping FIB pollution, e.g., [18].

Although detachment processes have been included in some existing FIB model structures, a current lack of information prevents good quality detachment representation across the spectrum of different faecal sources, especially non-agricultural sources, that often exist within a catchment [2,19]. For example, different livestock, wildlife and wildfowl excrete faecal material of varying physio-chemical characteristics, which in turn are likely to influence FIB mobilisation. Differences in faecal characteristics might include typical initial FIB concentrations, dry matter content and physical structure, all of which will change over time as a result of faecal ageing and, in the absence of rainfall, desiccation. Such changes will vary as a function of temperature and the combined effects of temperature and desiccation will impact on FIB survival [20]. Thus, temperature is likely to play a key role in influencing changes in FIB mobilisation potential of different faecal matrices over time.

FIB mobilisation from faecal sources is an important process but is often unaccounted for in tools and models designed to assess FIB risk in the environment [21]. There is also a lack of quantitative understanding of how FIB mobilisation from wildlife or wildfowl faeces may differ relative to common agricultural sources of faeces, which can lead landowners to query the FIB contribution from different sources [22]. The overarching aim of this study, therefore, was to determine whether there are differences in FIB mobilisation dynamics from a typical agricultural, wildlife and wildfowl source, namely Holstein-Friesian dairy cattle (*Bos taurus*), red deer (*Cervus elaphus*) and greylag goose (*Anser anser*) faeces. Specifically, our objectives were to: (i) quantify culturable FIB mobilisation from faecal sources following deposition; (ii) evaluate how two contrasting temperature conditions influence the temporal dynamics of culturable FIB mobilisation from faeces; and (iii) determine whether *E*. *coli* and intestinal enterococci (IE) exhibit differential mobilisation potential.

## Materials and methods

### Provenance of faeces used in all experiments

Fresh dairy faeces were collected from the livestock housing of a conventional dairy farm in Stirlingshire, Scotland. Cows were housed throughout the year and the farm operated a mechanical floor scraper in the barn meaning that older faecal material was removed, and the faeces collected was guaranteed to have been deposited within the previous 30 minutes. In total, ~12 dairy cow faecal deposits were collected and pooled. Fresh faeces of red deer were collected from the Scottish Deer Centre, Fife, Scotland. Red deer were selected as a representative wildlife species because they are widely distributed across much of Scotland and occupy a range of habitats that span moorlands through to woodlands [23]. The fields within which the deer were kept were harrowed prior to faecal collection, which ensured that all faeces collected were fresh (<12 h old). The diet of the deer used in this study was unlikely to have differed considerably from wild deer [1]. In total, ~30 faecal deposits from red deer were collected and pooled. Fresh faeces from greylag geese were collected from the Royal Society for Protection of Birds (RSPB) reservation located on the shores of Loch Leven, Fife. Greylag geese were selected as a representative wildfowl species because they are present year-round and are currently breeding successfully in several regions of Scotland [24]. The diet of the geese reflects that of a wild population. In total, ~50 faecal deposits from greylag geese were collected and pooled. After collection, all faeces were transferred immediately (< 1 h) to the laboratory for use in the experiment and thus no interim storage was required. No permits were required for collection of faecal samples because sampling was done with permission and assistance from the relevant land owners described above. Ethical approval for the project was granted by the University of Stirling General University Ethics Panel.

### Sampling of faecal material

A controlled laboratory experiment was carried out to mimic how rainfall mobilises FIB from faecal matter into the watercourses. Incubators (Sanyo Incubator MIR-153, Japan) were used to allow for two constant temperature treatments (0°C and 15°C) that represented two environmental scenarios under which faeces can be deposited: one a freezing scenario typical of winter conditions and the other typical of average summer temperature conditions in the UK [25]. The experiments were conducted over a duration of 12 days, allowing faecal material to age and dry. Every two days all faecal deposits were misted with sterile distilled water at a rate of 1 mL/100 cm^2^ of faecal surface area, as measured by the area of trays on which the samples were situated. This was done to mimic a ‘morning dew’ effect, and avoid complete dehydration of the faeces under incubator conditions [c.f. 26]. Each treatment consisted of five experimental replicates of faecal pat/pellet deposits that were destructively harvested per sampling day. The use of full-size dairy faecal deposits was impractical for a replicated laboratory experiment; therefore, faecal samples were bulked and homogenised in a sterile plastic container and then distributed into shallow circular 70 mm diameter foil trays as 50 g fresh weight dairy faecal deposits (84% moisture content). Likewise, deer faecal samples from several deer were pooled to form 50 g fresh weight piles of deer faecal pellets (78% moisture content) and goose faeces from several birds were pooled to form 10 g fresh weight goose faecal deposits (79% moisture content). The difference in mass used for geese faeces was due to the small amount of geese faeces excreted per day in the field relative to dairy cows and deer. Furthermore, using 50 g of goose faeces per experimental replicate was impractical because: (i) it was more important to guarantee freshness of faecal sample than volume of faecal sample, the latter being constrained by the size of the flock; and (ii) goose faeces are naturally much smaller, and such a volume was therefore not representative of typical goose faecal depositions. While the red deer and dairy cow samples used in the experiment were smaller than typical depositions in the field, such deposition piles are plausible if defecations occur while the animal is moving. In contrast, artificially increasing the size of a goose faecal deposit was considered unrealistic. Experimental replicates were randomly divided into each treatment and sampled on days 0, 3, 7 and 12 for use in the mobilisation experiment. This allowed for investigation of FIB release from freshly deposited faeces but also ageing faecal material held at either 0°C or 15°C. On sampling days, a total of 3 g was randomly sampled from each replicate of dairy and deer faeces using a sterile spatula. For goose faeces, the reduced starting faecal mass necessitated a smaller subsample of 1 g for use in the mobilisation experiment.

### Artificial sterile rainwater preparation

A standardised rainwater was prepared following the method described by [15]. The resulting rainfall (pH 5.64) had the following composition (g L^-1^): CaCl, 2.465; MgCl, 1.919; FeCl, 0.0445; NH4NO3, 0.430; K2SO4,0.617; NaCl, 3.317. The artificial rainwater was sterilised using an autoclave (15 min at 121°C) and maintained at 4°C prior to use.

### Simulating rainfall-initiated mobilisation

The DESPRAL test is a laboratory-based protocol originally developed to quantify phosphorus mobilisation from soil, with test results correlating well (r^2^ = 0.7–0.8) with amounts of suspended sediment and total phosphorus generated in overland flow using rainfall simulators (intensity 60 mm h^-1^ for 30 min) [27]. The DESPRAL approach was modified by Hodgson et al. [16] to evaluate FIB mobilisation from agricultural faecal matrices, and we have used this modified approach to quantify FIB mobilisation from an extended range of faecal sources. Briefly, 3 g of faeces was taken from each of the 5 replicate deer and dairy faecal samples for each time point and transferred to a 50 mL sterile centrifuge tube (*n* = 5) to which 27 mL of sterile standardised rainwater was pipetted slowly down the side of the tube to avoid disturbing the faeces. For goose faeces, 1 g was taken from each of the 5 replicate faecal samples for each time point and added to a 15 mL centrifuge tube (*n* = 5) to which 9 mL of sterile rainwater was added as described above. Different sized tubes were used to maintain as close a ratio as possible of liquid to air given the different mass of faeces; importantly, the faeces:rainwater ratios were consistent across all treatments (representing 1:10 dilutions). The tubes were mounted on a tabletop rotator, and rotated vertically (i.e., perpendicular to the benchtop) through 360^o^ for one minute at a speed of 35 revolutions per minute (rpm). This simulated a standardised interaction between faeces and rainfall, mimicking raindrop impact and subsequent faecal disruption. This approach provides an assay of FIB mobilisation potential under controlled laboratory conditions.

### FIB enumeration

To determine culturable counts of FIB, at each time point, 1 mL of eluent (i.e., ‘wash-off’) was transferred to 9 mL of sterile PBS and serial 10-fold dilutions were made using PBS. Briefly, 1 mL of each serially-diluted sample was pipetted on to a 0.45 μm cellulose acetate membrane and washed through a vacuum-filtration unit (Sartorius Stedim Biotech., Goettingen, Germany) with ~20 mL of sterile PBS. To determine presumptive *E*. *coli*, membrane filters were aseptically transferred to a Petri dish containing Membrane Lactose Glucuronide Agar (MLGA) (CM1031, Oxoid, Basingstoke, UK), inverted and incubated at 37°C (± 0.2°C) for 18–24 h. To quantify IE, membranes filters were aseptically transferred to Slanetz & Bartley medium (CM0377, Oxoid), inverted and incubated at 44°C (± 0.2°C) for 48 h. Following the method of Hodgson et al. [16], the remaining rainwater-faecal mix was homogenised by vortex mixing for 60 s and appropriate serial dilutions prepared again in sterile PBS. Duplicate FIB concentrations were determined for the faecal component, as described above for the eluent. This provided the basis for determining the mobilised fraction of FIB given that the total colony forming units (CFU) of FIB in the original sample could now be calculated. Method blanks (i.e., sterile PBS) were used to confirm aseptic technique and the flame sterilisation procedure between samples. The remaining faecal material from each replicate was used to determine the gravimetric water content by drying at 105°C for 24 h (until constant mass) and weighing the residual.

### Data analysis

All FIB counts underwent log_10_ transformation prior to statistical analysis, and distributions of CFU were log normally distributed as determined using the Kolmogorov−Smirnov goodness of fit test. Differences at the p < 0.05 level (95% confidence interval) were considered statistically significant. Proportions of cells mobilised from each faecal type were determined, although statistical analysis was performed on the actual CFU released per treatment and not on the proportion (%) of FIB mobilised. The proportion of CFU mobilised was calculated to account for the changes in source concentrations of FIB in faeces as a function of die-off and provided complementary data. A three-way analysis of variance (ANOVA) and a Tukey multiple comparison test were used to test for differences in FIB concentrations (over time at different temperatures from three faecal types). ANOVA was also used to test for differences in mobilisation of the two FIB and to determine whether there were differences in FIB concentrations in the different faecal types as the faeces aged. Student’s *t*-test was used to identify any difference between *E*. *coli* and IE concentrations within each faecal source. Pearson correlation coefficients were used to measure the strength and direction of relationships between moisture content of faeces and the percentage of FIB mobilised. All statistical analysis was performed using Minitab (Minitab 18.0 software, Minitab Inc.; State College, PA).

## Results

**Fig 1:**
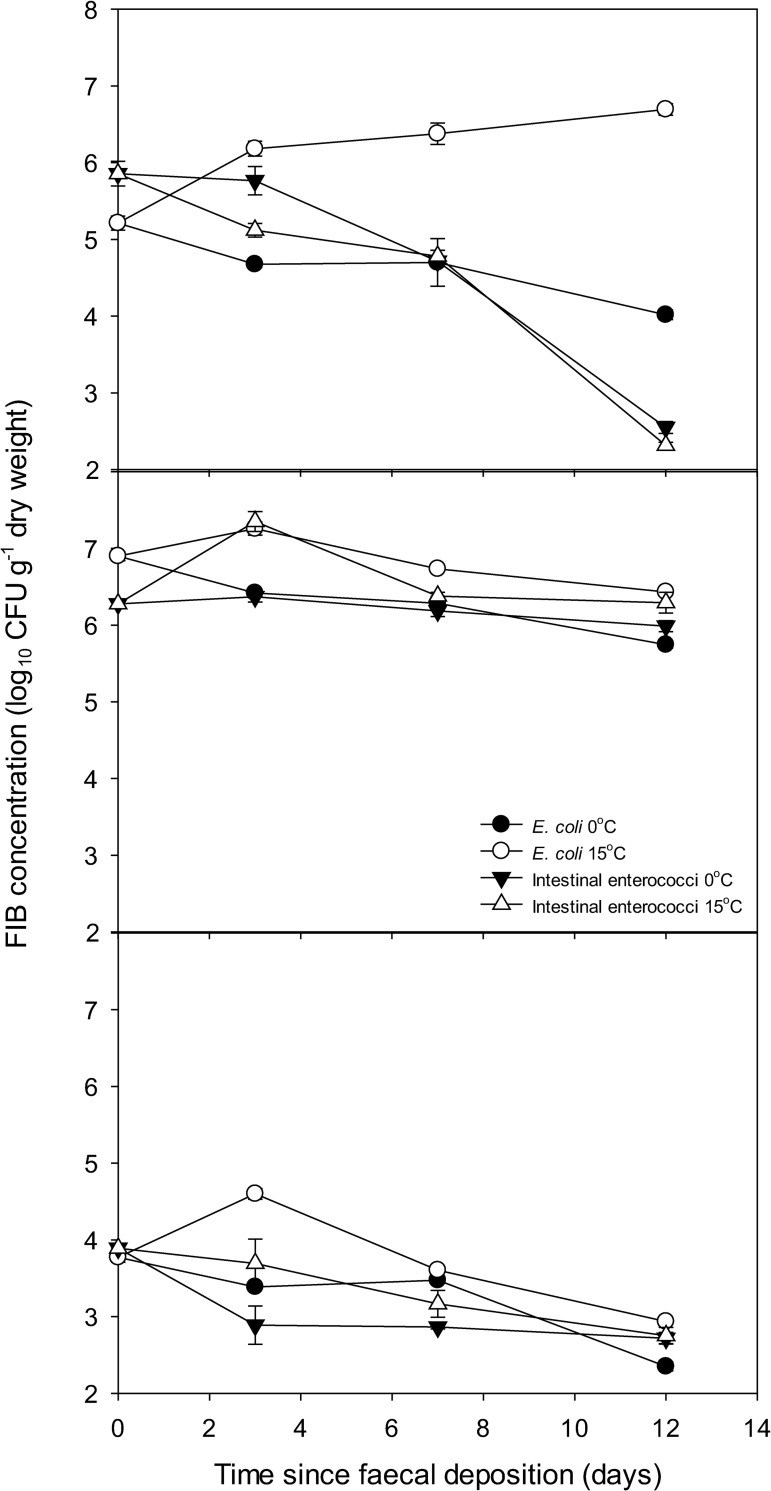
Persistence profiles of FIB in A) red deer faeces; B) dairy faeces; and C) greylag goose faeces. Data points are the mean of five replicates ± standard error.

All method blanks were negative for FIB indicating that no cross contamination occurred during sample processing. The persistence profiles of both *E*. *coli* and IE in all three faecal types, reported over time as CFU g^-1^ dry weight faeces, provide important contextual information to help understand proportions of FIB mobilised from the different faecal matrices (Fig 1). When excreted (day 0), concentrations of *E*. *coli* and IE in dairy faeces were significantly higher than concentrations found in deer faeces, which in turn were significantly higher than concentrations in goose faeces (P < 0.001; Fig 1). For *E*. *coli*, this pattern was consistent at all time points and for both temperatures apart from day 12 under 15°C, when cell concentrations were highest in deer faeces. The deer faeces incubated at 15°C and monitored for *E*. *coli* was the only scenario whereby the final concentration of FIB was higher than the starting concentration (P < 0.001). For IE, concentrations in deer faeces were lowest on day 12 when incubated at 15°C (P < 0.001). Earlier, on day 3, IE concentrations in deer and dairy were of a similar magnitude before concentrations in deer faeces dropped to levels consistent with goose faeces on day 7 (Fig 1). There were differences in relative proportions of the two FIB in fresh faeces: *E*. *coli* concentrations were significantly higher than IE concentrations in dairy faeces (P < 0.001); IE concentrations were significantly higher that *E*. *coli* concentrations in deer faeces (P < 0.01); and there was no significant difference between FIB concentrations in goose faeces (P > 0.05).

**Fig 2:**
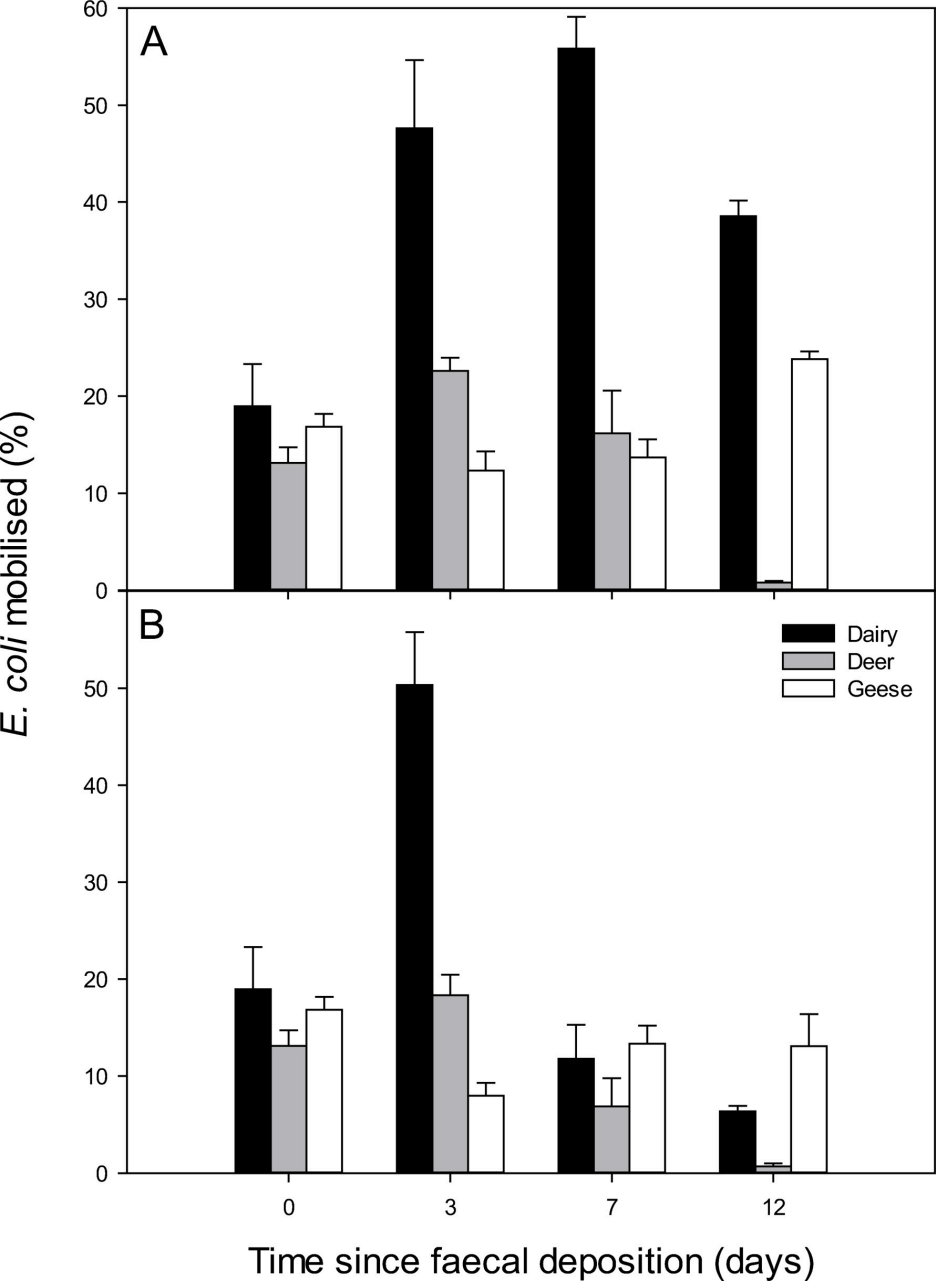
The proportion of *E*. *coli* mobilised from faeces over time. Faeces was stored under constant temperature conditions of A) 15°C and B) 0°C prior to each mobilisation assay. Data points are the mean of five experimental replicates ± standard error.

There were significant differences in the concentrations of both of the FIB mobilised from all three faecal types (P < 0.001). The order of mobilisation potential for both *E*. *coli* and IE from the faecal matrices (greatest to least, expressed as a proportion of the total present) was dairy cow > goose > deer. The proportion of *E*. *coli* mobilised relative to the source concentration was also determined (Fig 2). The mean *E*. *coli* concentration mobilised from dairy, deer and goose faeces was 6.01, 2.96 and 1.66 log_10_ CFU mL^-1^, respectively, when considering data from all time points post faecal deposition. For IE, the concentrations were 4.27, 1.96 and 1.24 log_10_ CFU mL^-1^, respectively. There were significant interactions between all factors, thus day and temperature had an interactive effect on FIB mobilisation as did day and faecal type and temperature and faecal type. Significantly higher concentrations of *E*. *coli* compared to IE were mobilised from dairy cow faeces (P < 0.05), deer faeces (P < 0.001) and goose faeces (P = 0.01).

*E*. *coli* concentrations recovered in the rainwater eluent identified significant changes in mobilisation potential over time from all faecal types. Mobilisation potential decreased significantly with time, with 12 days after deposition resulting in significantly less *E*. *coli* CFU per mL (P < 0.001). Mobilisation potential of *E*. *coli* was also influenced by the temperature at which the faecal material had aged over the course of the 12-day study (P < 0.001). In all cases, mobilised *E*. *coli* concentrations were greater when faeces were incubated at 15°C (Table 1).

**Table 1:** Total number of *E*. *coli* CFU in faecal and eluent samples, associated *E*. *coli* concentrations in eluent samples and percentage of cells mobilised for each time-point. Data are the mean of five replicates (± standard error).

Faecal matrix	Day	Total *E*. *coli* in faecal sample (Log_10_ CFU)		Total *E*. *c*o*li* in eluent (Log_10_ CFU)		*E*. *coli* concentration in eluent (Log_10_ CFU mL^-1^)		*E*. *coli* mobilised at point in time (%)	
		15°C	0°C	15°C	0°C	15°C	0°C	15°C	0°C
Dairy	0	6.58 (0.07)	6.58 (0.07)	5.82 (0.13)	5.82 (0.13)	4.39 (0.13)	4.39 (0.13)	18.96 (4.34)	18.96 (4.34)
	3	7.26 (0.10)	6.30 (0.05)	6.92 (0.12)	5.99 (0.04)	5.49 (0.12)	4.56 (0.04)	47.63 (7.01)	50.34 (5.44)
	7	7.03 (0.02)	6.46 (0.09)	6.77 (0.04)	5.41 (0.13)	5.34 (0.04)	3.97 (0.13)	55.83 (3.29)	11.79 (3.50)
	12	6.83 (0.01)	6.15 (0.03)	6.41 (0.03)	4.94 (0.04)	4.98 (0.03)	3.51 (0.04)	38.55 (1.62)	6.35 (0.56)
Deer	0	5.14 (0.09)	5.14 (0.09)	4.24 (0.11)	4.24 (0.11)	2.82 (0.11)	2.82 (0.11)	13.12 (1.60)	13.12 (1.60)
	3	6.30 (0.09)	4.86 (0.03)	5.65 (0.11)	4.11 (0.08)	4.23 (0.11)	2.69 (0.08)	22.60 (1.38)	18.35 (2.10)
	7	6.82 (0.12)	5.17 (0.31)	5.96 (0.20)	3.78 (0.09)	4.53 (0.20)	2.36 (0.09)	16.17 (4.40)	6.86 (2.92)
	12	7.21 (0.07)	4.52 (0.07)	5.03 (0.19)	2.01 (0.29)	3.61 (0.19)	0.58 (0.29)	0.79 (0.17)	0.70 (0.31)
Goose	0	3.77 (0.07)	3.77 (0.07)	2.99 (0.09)	2.99 (0.09)	2.04 (0.10)	2.04 (0.09)	16.84 (1.33)	16.84 (1.33)
	3	4.60 (0.07)	3.39 (0.02)	3.67 (0.09)	2.26 (0.06)	2.71 (0.09)	1.31 (0.06)	12.32 (1.98)	7.98 (1.30)
	7	3.60 (0.02)	3.47 (0.02)	2.72 (0.04)	2.58 (0.05)	1.77 (0.04)	1.63 (0.05)	13.68 (1.86)	13.34 (1.86)
	12	2.94 (0.02)	2.35 (0.06)	2.31 (0.01)	1.36 (0.21)	1.36 (0.01)	0.41 (0.21)	23.81 (0.80)	13.10 (3.28)

**Fig 3:**
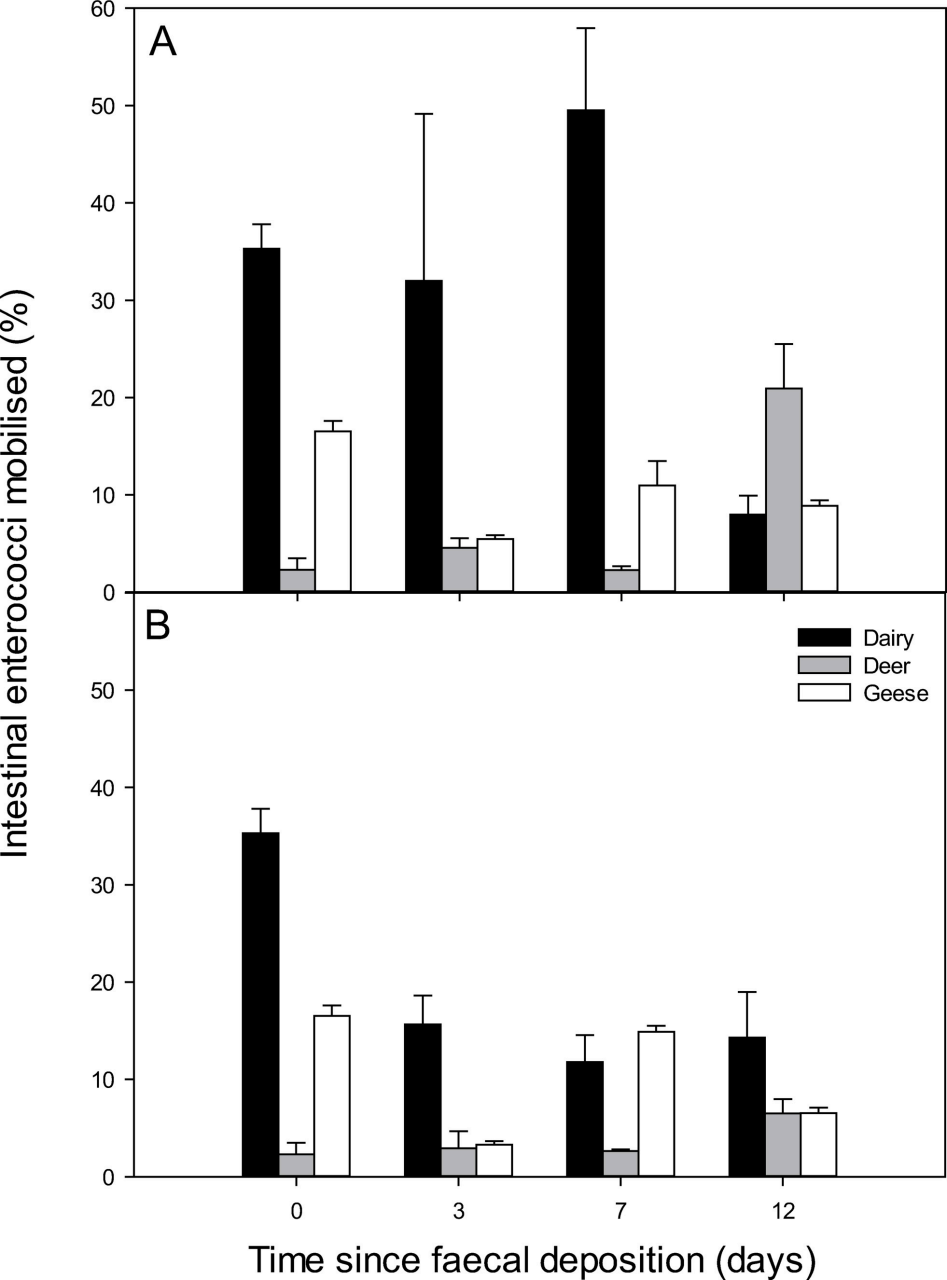
The proportion of intestinal enterococci mobilised from faeces over time. Faeces was stored under constant temperature conditions of A) 15°C and B) 0°C prior to each mobilisation assay. Data points are the mean of five experimental replicates ± standard error.

**Fig 4:**
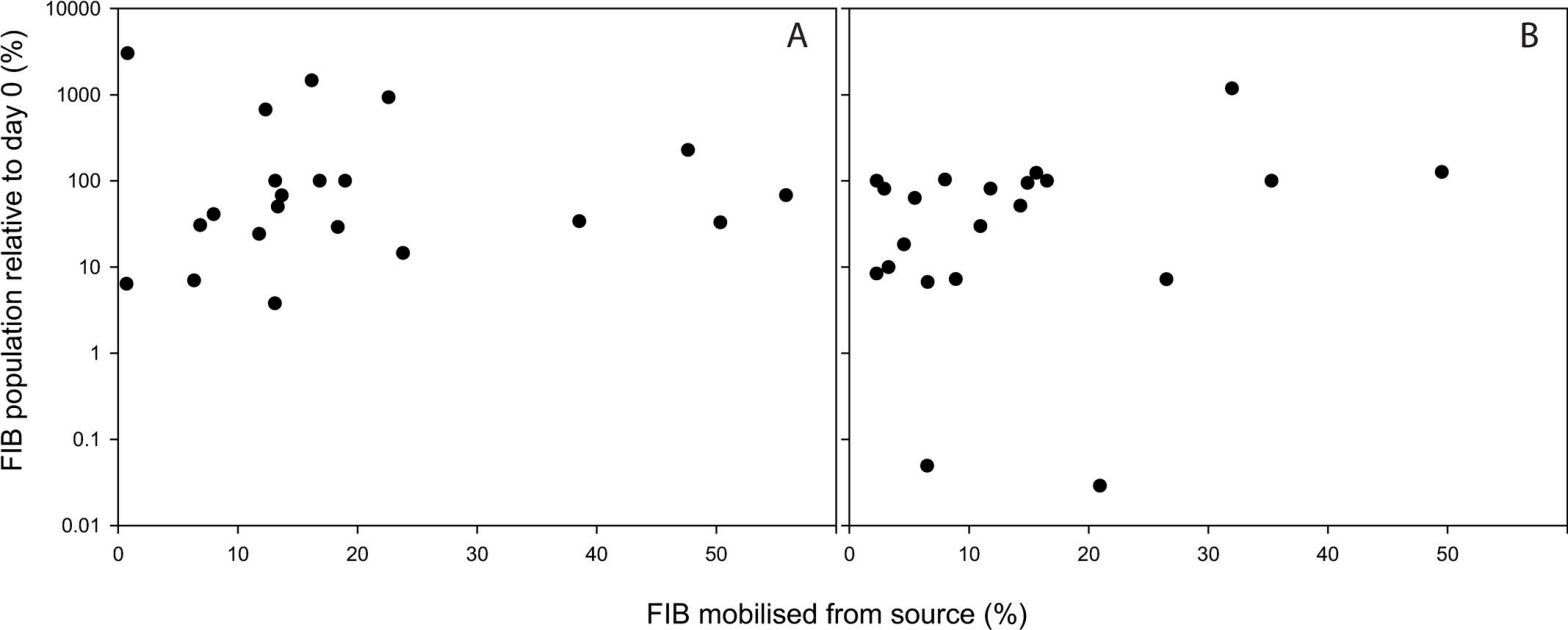
The proportion of *E*. *coli* (A) and intestinal enterococci (B) mobilised as a function of the FIB source load for all three faecal sources combined (normalised to reflect the FIB population relative to day 0.

Temporal patterns of IE mobilisation varied from that of *E*. *coli*; concentrations of IE recovered in rainwater from dairy faeces were not significantly different over time (P > 0.05). There were, however, significant differences in IE concentrations lost from deer (day 0 and 3 > day 7 > day 12) and goose (day 0 > day 3, 7 and 12) faeces over time. The warmer temperature treatment promoted significantly higher mobilisation of IE concentrations from dairy faeces (P < 0.001) but no temperature-driven mobilisation effect was observed for IE from deer or goose faeces (P > 0.05) (Table 2). Despite no difference in mobilised IE concentrations in dairy faeces over time, Fig 1 shows that IE concentrations in all three faecal sources fluctuated over the course of the experiment and in turn influenced the proportion of IE that was mobilised (Fig 3). The concentration and the proportion of mobilised FIB therefore represent two different measures of mobilisation. No clear relationship between *E*. *coli* or IE mobilisation as a function of the FIB source load (normalised to reflect the FIB population relative to day 0) was observed across all faecal sources and timepoints (Fig 4A and 4B).

**Table 2:** Total number of intestinal enterococci CFU in faecal and eluent samples, associated intestinal enterococci concentrations in eluent samples and percentage of cells mobilised for each time-point. Data are the mean of five replicates (± standard error).

Faecal matrix	Day	Total intestinal enterococci in faecal sample (Log_10_ CFU)		Total intestinal enterococci eluent (Log_10_ CFU)		Intestinal enterococci concentration in eluent (Log_10_ CFU mL^-1^)		Intestinal enterococci mobilised at point in time (%)	
		15°C	0°C	15°C	0°C	15°C	0°C	15°C	0°C
Dairy	0	5.96 (0.04)	5.96 (0.04)	5.50 (0.06)	5.50 (0.06)	4.07 (0.06)	4.07 (0.06)	35.29 (2.52)	35.29 (2.52)
	3	7.35 (0.15	6.25 (0.05)	6.54 (0.19)	5.41 (0.08)	5.11 (0.19)	3.98 (0.08)	31.99 (17.16)	15.63 (2.96)
	7	6.67 (0.06)	6.36 (0.08)	6.34 (0.03)	5.36 (0.08)	4.91 (0.03)	3.92 (0.08)	49.52 (8.44)	11.80 (2.74)
	12	6.69 (0.14)	6.39 (0.07)	5.53 (0.18)	5.44 (0.13)	4.10 (0.18)	4.01 (0.13)	7.99 (1.95)	14.30 (4.67)
Deer	0	5.78 (0.16)	5.78 (0.16)	3.97 (0.11)	3.97 (0.11)	2.55 (0.11)	2.55 (0.11)	2.29 (1.19)	2.29 (1.19)
	3	5.23 (0.09)	5.95 (0.19)	3.86 (0.03)	4.19 (0.05)	2.44 (0.03)	2.77 (0.05)	4.57 (0.99)	2.92 (1.77)
	7	5.22 (0.06)	5.18 (0.07)	3.56 (0.04)	3.60 (0.07)	2.13 (0.04)	2.18 (0.07)	2.28 (0.40)	2.65 (0.16)
	12	2.83 (0.04)	3.06 (0.06)	2.09 (0.11)	1.81 (0.07)	0.67 (0.11)	0.39 (0.07)	20.94 (4.57)	6.50 (1.48)
Goose	0	3.89 (0.01)	3.89 (0.01)	3.11 (0.03)	3.11 (0.03)	2.15 (0.03)	2.15 (0.03)	16.52 (1.07)	16.52 (1.07)
	3	3.69 (0.32)	2.89 (0.25)	2.43 (0.34)	1.39 (0.20)	1.47 (0.34)	0.44 (0.20)	5.47 (0.39)	3.27 (0.38)
	7	3.17 (0.17)	2.87 (0.03)	2.12 (0.13)	2.04 (0.01)	1.167 (0.13)	1.08 (0.01)	10.96 (2.51)	14.89 (0.62)
	12	2.75 (0.11)	2.72 (0.08)	1.85 (0.08)	1.53 (0.10)	0.90 (0.08)	0.57 (0.10)	8.89 (0.55)	6.54 (0.57)

**Fig 5:**
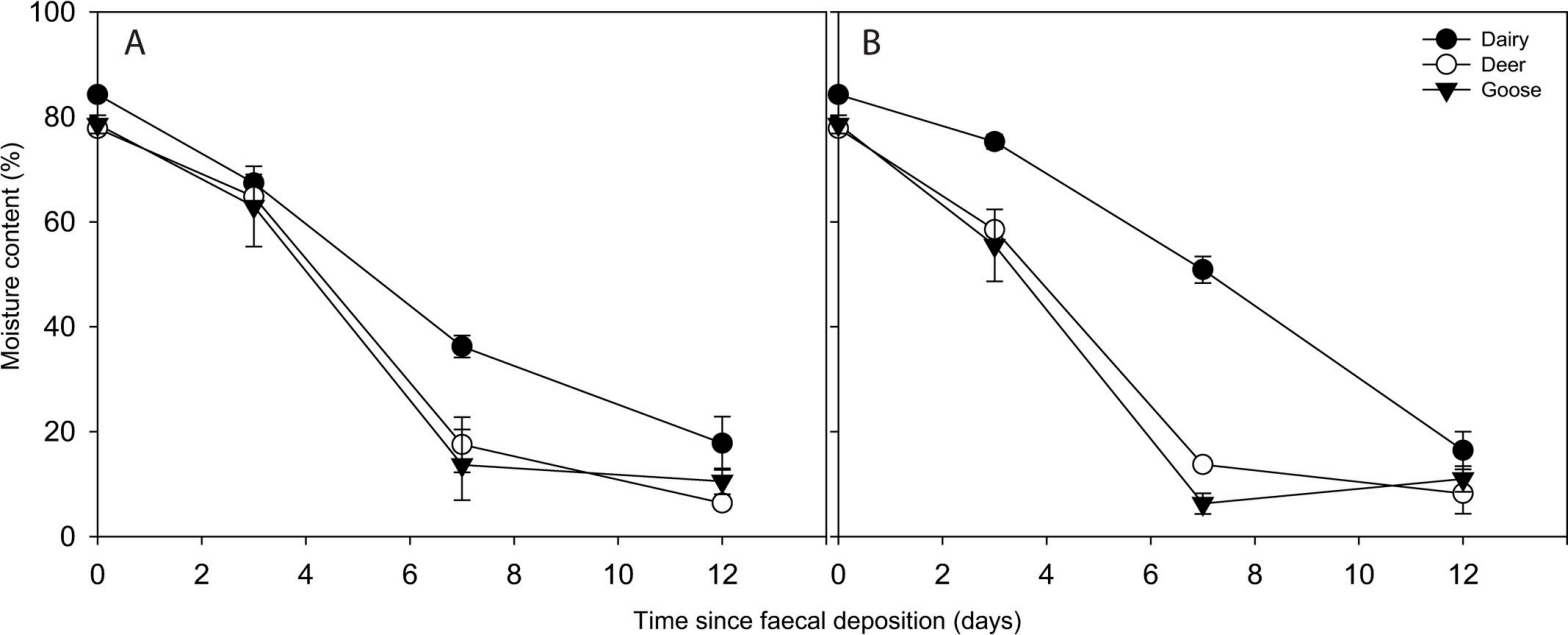
Change in moisture content of faeces over time when held at a constant 15°C (A) and 0°C (B). Data points are the mean of five replicates ± standard error.

Changes in moisture content of the three faecal sources were evident over the duration of the experiment (Fig 5). Deer and goose faeces showed similar patterns of moisture loss under both temperature treatments. Dairy faeces retained more moisture over the duration of the experiment, with the difference in moisture content in dairy faeces relative to deer and goose faeces most prominent on day 7 under both temperature treatments. At a temperature of 15°C, the change in moisture content over the experiment represented a decrease of 71.4%, 68.0% and 66.5% for deer, goose and dairy faeces, respectively. The overall change in moisture content when faeces were held at 0°C was similar, with a decrease of 69.6%, 67.9% and 67.8% moisture recorded for deer, goose and dairy faeces, respectively. No significant correlation was observed between faecal moisture content and FIB mobilisation from dairy cow or goose faeces. In deer faeces, a significant positive correlation (r = 0.66, P < 0.001) and significant negative correlation (r = 0.41, P < 0.05) was recorded between moisture content of the faeces and the percentage of E. coli and intestinal enterococci mobilised, respectively.

## Discussion

The potential for FIB mobilisation from faecal material varies with time, temperature and the source of faecal material. Patterns of mobilisation are different for the two most common FIB, *E*. *coli* and IE; however, quantifying mobilisation of microorganisms from faecal matrices is complex because of the dynamic nature of microbial persistence patterns, which again can differ for *E*. *coli* and IE. Mobilisation therefore reflects a combination of FIB survival in the faeces versus the erodibility of the faecal source and subsequent detachment rate of the FIB population, which both vary as a function of faecal ageing and desiccation [10]. Detachment processes are the main driver of FIB mobilisation from faeces, although mechanistic understanding of how different faecal sources influence FIB retention and mobilisation is limited [28]. In our study we specifically quantified FIB mobilisation from three different faecal matrices and contribute important process-based information concerning how FIB mobilisation changes over time and under different temperature scenarios.

The FIB concentrations mobilised from dairy faeces after 12 days, was substantially higher than from red deer and greylag goose faeces and reflects the varying levels of FIB present in the source of the faeces, with goose faeces containing much lower concentrations of FIB overall in our study. The one exception was the concentration of *E*. *coli* mobilised from deer faeces after 12 days; however, the *E*. *coli* population in deer faeces at 15°C underwent considerable regrowth and deviated substantially from the persistence profiles of other faecal sources leading to a greater availability of *E*. *coli* for mobilisation. Regrowth of FIB in faecal material is not uncommon, and over an order of magnitude increase in *E*. *coli* numbers post excretion has been reported in deer faecal pellets incubated at lower temperatures than those under which regrowth was observed in our study (4°C versus 15°C) [29]. The magnitude of decline in concentration of mobilised FIB was greatest for goose faeces, whereas in general the other faecal sources maintained similar levels to day 0, suggesting a more consistent risk of FIB release to the environment from dairy cow and deer faeces over the study duration. Mobilisation potential of FIB will vary depending on the starting concentration of FIB at source, but more research is needed to fully characterise FIB mobilisation at the point of excretion, over time, and through different seasons, which is important given the potential for large variability in FIB shedding rates from different animals [26]. Prior research has acknowledged the challenges that a dynamic FIB population can introduce as part of quantifying mobilisation, both in terms of FIB regrowth and via FIB decay [30].

When considering the proportion of FIB mobilised relative to the source load, rather than mobilised FIB concentrations, dairy cow faeces still consistently generated greater *E*. *coli* mobilisation from the faecal matrix relative to the other faecal sources under the warmer temperature treatment. The dominance of the livestock faecal source in generating greater proportions of mobilised *E*. *coli* relative to the wildlife / wildfowl faecal sources was more short-lived at 0°C; a pattern repeated in the IE mobilisation data too. To some extent this will be due to variations in survival of FIB at the two temperatures investigated, with freezing temperatures known to be less conducive to FIB persistence [1]. While a proportion of cells may have subsequently transitioned into a viable-but-non-culturable state in some treatments [31], the mobilisation parameters reported here remain crucially important given that models used to inform on FIB fate and transfer, and guide landscape decision-making, are largely built on data derived from culturable counts in order to align with culture-based standards used by environmental regulators [1]. However, changes in mobilisation will also be influenced by how quickly the outer layers of the faecal matrix develop a crust in the absence of rainfall. As faecal matter aged under constant temperatures, the moisture content of the faecal matrix also decreased. This was more rapid for the red deer and greylag goose faeces than for dairy cow faeces, which would have retained a moist interior for longer due to differences in surface area to volume ratio and offers further explanation for the more readily available supply of cells in the dairy faeces for mobilisation. Beef cow faeces monitored previously using the same experimental approach also demonstrated greater mobilisation potential relative to other manure sources, suggesting that the physical make-up of cowpats is conducive for releasing substantial numbers of FIB even with lengthy lag times between faecal deposition and the onset of rainfall [16].

Concentrations of mobilised *E*. *coli* were substantially less after 12 days relative to earlier in the experiment in all faecal sources, while for IE this was only the case for goose and deer faeces. Under the colder temperature regime, FIB concentrations declined but remained orders of magnitude above detection limits in all faecal sources; however, mobilised concentrations were further reduced, and this may reflect limited mobilisation potential arising from the formation of a crust rather than reduced FIB survival alone [32]. Under field conditions, a crust forms when faeces are exposed to sunlight [33], but under laboratory conditions the constant temperature and lack of rehydration from rainfall would help to promote more rapid drying in the faecal boundary layers, and thus a dry ‘skin’ on the faeces surface likely developed that acted as a form of crust. The misting step done to mimic morning dew formation would not compensate for the level of rehydration provided by rainfall, nor was this the intention.

The deer, dairy and goose faeces represent different faecal matrix structures and the differences in the rate of FIB mobilisation from them are probably related to the differences in physical structure and make-up of the faecal sources. Differences in physical structure of deer and dairy faeces can also impact FIB survival rates [1], while the soluble/solid faecal composition can govern the release rates of FIB to the wider environment [10,28]. The faecal structure will also dictate the speed at which infiltrating water can make contact with resident cells, in turn facilitating their wash-out [11]. The proportion of finer and larger fractions of organic matter that characterises each faecal matrix may also explain differences in mobilisation potential, as has been reported in studies exploring *E*. *coli* release from specific fractions of faeces (0.25, 0.5, 1.0, 2.0 mm faecal components) [34]. FIB mobilisation will be influenced by the degree of erodibility associated with the faecal source [11] and observations of faecal disruption during the DESPRAL test identified that dairy faeces more readily disaggregated relative to the goose and deer faeces. The pellet-like structure of deer and, to a lesser extent, goose faeces is similar to sheep faecal pellets, with the latter also demonstrating an ability to maintain its physical composition when subjected to the same experimental approach [16]. This suggests that FIB mobilisation patterns from wildlife and wildfowl faeces are not distinct from a number of other livestock FIB sources, e.g. sheep faeces and farmyard manures, but that larger faecal pats associated with cattle present greater risk of contributing FIB to hydrological pathways with the onset of rainfall. Information on the chemo-physical (e.g., fibre content) and main organic composition of samples was not collected in this study. Such analysis could provide useful data to further interpretate the results and aid comparisons with studies of other animal faecal sources. Therefore, future research should include such supporting information if possible.

The results of our study highlight changes in FIB mobilisation as a function of increasing lag time between faecal excretion and rainwater contact with the faecal source. The role of subsequent rewetting episodes and how consecutive rainfall events influence mobilisation was not considered. Wetting and drying cycles are likely to be influential in altering mobilisation potential and requires further research. Recent studies have highlighted the importance of freeze-thaw processes on FIB survival [1] and like wetting and drying cycles the physical changes to faecal structure resulting from freeze-thaw cycles will probably lead to changes in mobilisation potential of FIB from faecal sources. Few studies are available that specifically quantify how rainfall recurrence impacts on FIB mobilisation from a suite of faecal sources. There are examples of larger scale monitoring campaigns that consider the impacts of repeated rainfall events across multiple landscape sources on FIB export via drainage networks, but again studies such as this are specifically quantifying FIB transfer and delivery and not mobilisation *per se*, e.g., [35].

Under field conditions, the duration of a rainfall event will influence FIB mobilisation dynamics and the two key detachment processes operating over the ‘event’ are likely to be raindrop impact and subsequent sloughing of the faecal surface in response to resulting surface runoff, in turn leading to the gradual disintegration of the faecal matrix. A number of factors will influence the intensity of raindrop impact and subsequent cell detachment and these include the kinetic energy of the falling precipitation, the angle of raindrop contact and the moisture content of the material being impacted by the raindrops [36]. In our study the ‘event’ was limited in duration to 60 s and the disruption to the faecal source was constant over that timeframe because we used a controlled laboratory assay specifically developed to investigate FIB mobilisation [15]. We did not determine how FIB mobilisation varied over the duration of the ‘event’. Over longer experimental timeframes and using different measures of mobilisation there are reports of a faster, or in some cases more irregular, initial FIB release from faeces followed by a slower steady-state FIB release [34,37]. Despite a smaller mass of faeces being used in assessing FIB mobilisation from goose faeces, the ratio of faecal mass to rainwater was consistent with that used to determine FIB concentrations released from dairy cow and deer faeces, and thus the difference in faecal mass should not impact on comparing the concentrations of FIB mobilised across different treatments.

Differences in mobilisation characteristics of *E*. *coli* and IE probably reflected varying properties of the different bacterial cells, e.g., physiological properties, surface structure, that can influence mechanisms associated with their release from liquid versus solid fractions of the faecal matrix [14]. For example, it has been suggested that *E*. *coli* resides in the more liquid fraction of manure whereas IE associates with more strongly with particles and this can impact the relative release dynamics following rainfall detachment [38]. The proportions of FIB mobilised from the three faecal sources were of a similar magnitude to those reported by Hodgson et al., [16] who used the same methodology (but at a slower, less intense DESPRAL test rpm) to mimic rainfall driven mobilisation. In general, faecal sources with higher moisture content (beef cow faeces, dairy cattle slurry [16]; dairy cow faeces; (this study)) were found to promote mobilisation of up to ~ 50% of the FIB in the faeces at times of peak mobilisation, whereas faecal sources with lower moisture content (sheep faeces, farmyard manure [16]; deer and goose faeces (this study)) tended to promote up to ~ 20% mobilisation. However, the dynamic relationship between changes in moisture content of faeces and mobilisation of FIB is more complicated, as evidenced by no clear correlation between these variables in dairy cow or goose faeces and with inverse relationships observed between moisture content and *E*. *coli* versus intestinal enterococci mobilisation from deer faeces over the experiment duration. This complication arises because of the combined influence of both die-off and regrowth on FIB persistence. It is worth highlighting that the DESPRAL test, originally developed to estimate the intrinsic risk of sediment and phosphorus mobilisation from a wide range of bare European soils [27], is a laboratory-based simulation that provides a surrogate for assessing rainfall-driven mobilisation rather than a direct measure of, for example, rain-drop impact detachment processes. Our application of the DESPRAL test for measuring FIB mobilisation from faecal sources provides an assay for the relative likelihood of FIB detachment following interaction with rainwater. The approach has been used widely as a proxy for pollutant mobilisation [e.g., 16,39–41].

Data reported in our study can help to constrain the parameterisation of mobilisation coefficients in models, which are often ignored in the modelling of FIB fate and transfer because of a lack of such information [2,19]. Understanding mobilisation potential is important because it provides an indication of the magnitude of a pollutant load that may subsequently be transferred through the environment. The inclusion of laboratory-derived mobilisation coefficients into a landscape model would, however, require careful assessment but this would be the case for any laboratory-derived process representation [1]. While the mobilisation data generated from the DESPRAL experiments does not include molecular analyses it is important to highlight that the majority of models used to understand FIB pollution are developed on data derived from culture-based studies [42].

Published studies report on FIB mobilisation, but also FIB release and/or removal, and there is some ambiguity in the use of terminology in studies reporting on FIB loss from faeces. Blaustein et al [10] recognise two meanings associated with the term release: in some studies, this is used to represent FIB leaving a faecal matrix, whereby release is a boundary condition, but in other studies release is taken to mean cell concentrations found within runoff and leachate at plot scales or coarser. The latter, in our view, is not truly a measure of mobilisation because some cells that are mobilised from the faecal source may become trapped on the soil surface or in the soil pore architecture prior to sample collection. FIB concentrations measured in runoff and leachate represent FIB in a state of transfer through the environment. They have already been mobilised from the faeces by detachment processes such as raindrop impact and sloughing. The data reported in our study therefore provides new information relating specifically to mobilisation rates of FIB from three common faecal sources in rural catchments. The findings will enable refinement of existing models and decision support tools that recognise detachment processes as an important step in understanding FIB risk to the wider environment.

## Conclusion

The loss of FIB from land to water from diffuse sources represents a continuum whereby cells are mobilised from faeces following rainfall and transferred in hydrological pathways before being delivered to a receiving water. Our findings add quantitative evidence to support our understanding of FIB mobilisation potential from three important faecal sources in the environment and demonstrate that dairy cow faeces represent a greater risk of contributing FIB to the wider environment than goose or deer faeces following rainfall events. As faeces age, deer and geese contributions can become more important contributors of mobilised FIB, highlighting a complex and nuanced pattern to FIB mobilisation from different sources typical of rural catchments. While our study focused on mimicking hydrological mobilisation from the terrestrial environment, such processes can be circumvented if faecal deposition by livestock, wildlife or wildfowl occurs direct to a receiving water and the rate of faecal breakdown and dissolution as drivers of FIB mobilisation following submergence would then require investigation. Our findings provide novel data to help characterise this important, yet often underrepresented process associated with FIB fate and transfer; however, further laboratory and field quantification of mobilisation processes is needed to support the parameterisation of modelling efforts designed to predict FIB impairment of receiving waters in catchments that contain complex mixes of faecal sources.
